# Tandem autologous stem cell transplant in multiple myeloma patients with minimal residual disease: an explorative study

**DOI:** 10.1007/s44313-025-00101-6

**Published:** 2025-10-23

**Authors:** Sieun Oh, Sung-Soo Park, Jung Yeon Lee, Jae-Ho Yoon, Sung-Eun Lee, Hee-Je Kim, Seung-Hwan Shin, Young-Woo Jeon, Seung-Ah Yahng, Jin Jung, Ari Ahn, Myungshin Kim, Chang-Ki Min

**Affiliations:** 1 Department of Hematology, Seoul St. Mary’s Hospital, College of Medicine, The Catholic University of Korea, Seoul, Republic of Korea; 2 Catholic Research Network for Multiple Myeloma, Seoul, Republic of Korea; 3 Catholic Hematology Hospital, Seoul St. Mary’s Hospital, College of Medicine, The Catholic University of Korea, Seoul, Republic of Korea; 4 Myeloma Center, Eunpyeong St. Mary’s Hospital, The Catholic University of Korea, Seoul, Republic of Korea; 5 Department of Hematology, Yeouido St. Mary’s Hospital, College of Medicine, The Catholic University of Korea, Seoul, Republic of Korea; 6 Department of Hematology, Incheon St. Mary’s Hospital, College of Medicine, The Catholic University of Korea, Incheon, Republic of Korea; 7 Department of Laboratory Medicine, Incheon St. Mary’s Hospital, College of Medicine, The Catholic University of Korea, Incheon, Republic of Korea; 8 Department of Laboratory Medicine, College of Medicine, The Catholic University of Korea, Seoul, Republic of Korea; 9 Catholic Genetic Laboratory Center, Seoul St. Mary’s Hospital, College of Medicine, The Catholic University of Korea, Seoul, Republic of Korea

**Keywords:** Multiple myeloma, Minimal residual disease, Tandem autologous stem cell transplantation

## Abstract

**Purpose:**

Tandem autologous stem cell transplantation (tASCT) is a viable option for high-risk multiple myeloma (MM) patients. Minimal residual disease (MRD), a real-time surrogate marker of disease burden, serves as a valuable measure of treatment response. This study evaluated the impact of tASCT on MRD dynamics in MM patients.

**Methods:**

We analyzed data from a multicenter registry of 28 patients who underwent tASCT as frontline treatment between January 2019 and October 2024. Eligibility criteria included undergoing two ASCTs within one year, having MRD positivity before tASCT, and completing follow-up MRD assessment. Patients were stratified into two groups: extensive MRD clearance (≥ 50% reduction, *n* = 18) and modest MRD clearance (< 50% reduction, *n* = 10).

**Results:**

Across the entire cohort, mean MRD decreased from 0.111% pre-tASCT to 0.056% post-tASCT. Three patients achieved MRD negativity, 20 had reductions without negativity, and five experienced increases. The extensive clearance group showed significant MRD reduction (0.152% to 0.017%) and longer progression-free survival (PFS: 37.7 vs. 16.3 months, *p* = 0.013) compared with the modest clearance group, in which MRD increased (0.175% to 0.830%). Overall survival did not differ significantly.

**Conclusions:**

tASCT provides clinical benefit for MRD-positive MM patients, particularly those achieving significant MRD reduction. These findings support tASCT as a feasible approach for MRD-positive patients following initial ASCT.

## Introduction

Multiple myeloma (MM) is a heterogeneous hematologic malignancy characterized by clonal plasma cell proliferation [1]. Despite significant advances in treatment, MM remains incurable for most patients, underscoring the need for personalized therapeutic approaches and continuous response monitoring to guide subsequent treatment decisions. The International Myeloma Working Group has proposed complete response (CR) and stringent complete response (sCR) as major therapeutic goals in MM [2]. While these criteria are central to clinical practice, their ability to predict long-term outcomes remains limited. Studies comparing sCR and CR have produced mixed results, highlighting the need for more sensitive methods to capture the dynamic nature of MM and to guide treatment more effectively [3, 4].

In this context, minimal residual disease (MRD) has emerged as a crucial tool in evaluating treatment response, capable of detecting residual myeloma cells at a sensitivity as low as 10^–5^. [5, 6]. MRD positivity has consistently been associated with poor prognosis, making it an essential target in treatment strategies aimed at achieving long-term disease control [7, 8]. Incorporating MRD assessment into clinical practice provides a more precise approach to post-treatment decision-making, particularly for consolidation and maintenance therapies [9]. Notably, MRD status evaluated three months post-treatment has shown a significant correlation with improved survival outcomes [10–12], reinforcing its potential role as a surrogate marker of therapeutic efficacy.

Autologous stem cell transplantation (ASCT) is considered a standard component of frontline treatment for transplant-eligible patients [13]. Additionally, tandem ASCT (tASCT)—the performance of two sequential ASCT procedures—has demonstrated significant benefits in MM patients, particularly those with cytogenetically high-risk profiles [14–16]. While traditional risk factors, such as cytogenetic abnormalities, provide valuable baseline prognostic information, they fail to capture the evolving nature of the disease during treatment. Integrating MRD status with tASCT may offer a more dynamic method of assessing treatment efficacy and guiding subsequent therapeutic decisions.

In this exploratory study, we aim to investigate the potential of combining MRD assessment with tASCT in MM patients. By stratifying patients based on MRD dynamics, we seek to demonstrate how MRD-guided strategies can refine treatment outcomes and ultimately pave the way for more personalized therapeutic approaches in MM.

## Methods

### Patient selection

**Fig. 1:**
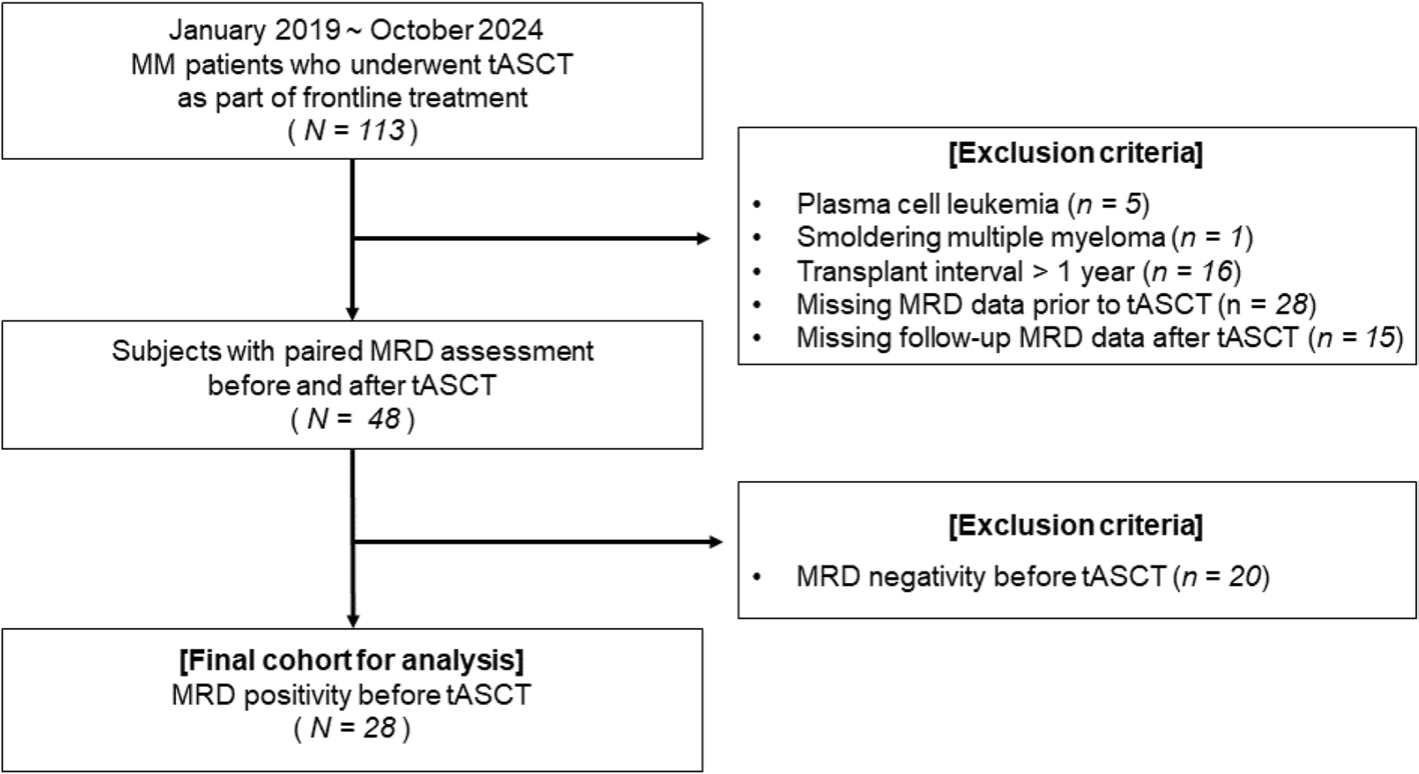
Construction of the study cohort. *Abbreviations*: MM, multiple myeloma; tASCT, tandem autologous stem cell transplantation; ASCT, autologous stem cell transplantation; MRD, minimal residual disease

Since January 2019, we have recommended tandem ASCT for patients who underwent the first ASCT and met at least one of the following criteria: (i) age < 60 years, (ii) persistent MRD positivity after the first ASCT, (iii) achievement of only a partial response (PR) after the first ASCT [2], (iv) presence of at least one high-risk cytogenetic abnormality (such as del(17p), t(4;14), t(14;16), or 1q gain/amplification), and/or (v) Revised Multiple Myeloma International Staging System stage III at diagnosis [17]. Nonetheless, despite meeting these criteria, some patients did not proceed with tASCT because of physician judgment regarding frailty during the observation period or patient refusal related to concerns about toxicity or socioeconomic constraints. We initially identified 113 consecutive MM patients who underwent tASCT as part of frontline treatment between January 2019 and October 2024 using data from the multicenter Catholic Research Network for Multiple Myeloma (CAREMM) registry. A total of 65 patients were excluded based on the following criteria: (i) diagnosis of plasma cell leukemia (*n* = 5) or smoldering multiple myeloma (*n* = 1), (ii) an interval exceeding one year between sequential transplantations (*n* = 16), and (iii) lack of MRD data following either the first ASCT (*n* = 28) or tASCT (*n* = 15). Additionally, 20 patients who achieved MRD negativity before tASCT were excluded. As a result, the final study cohort comprised 28 patients who exhibited MRD positivity within one year of the initial ASCT and subsequently received tASCT (Fig. 1). Ethical approval for this study was obtained from the Institutional Review Board of Seoul St. Mary’s Hospital (No. KC24RIDI0685). Because this study involved a retrospective analysis of existing data, the requirement for informed consent was waived.

### MRD measurement

MRD assessments were conducted within one year after both the initial ASCT and tASCT. MRD was measured using the DURAClone multiparametric flow cytometry (MFC) protocol (Beckman Coulter), which has an analytical sensitivity of 1 × 10^–5^, as demonstrated in our previous research [7]. The DURAClone panel used in this study included eight antibodies targeting MM-specific plasma cells: CD81-FITC, CD27-PE, CD19-PC5.5, CD200-PC7, CD138-APC, CD56-APC-A750, CD38-PB450, and CD45-KO525. Data were analysed using Kaluza software, version 2.1 (Beckman Coulter), ensuring precise interpretation of flow cytometry results. The assay’s limit of detection was 6 × 10^–6^ and the lower limit of quantification was 1 × 10^–5^. MRD positivity was defined according to the limit of detection, applying a stringent threshold for reliable MRD detection [7].

### Procedure of frontline treatment

Induction regimens included four to six cycles of bortezomib-thalidomide-dexamethasone (VTd), bortezomib-lenalidomide-dexamethasone (VRd), or VTd combined with daratumumab (DVTd). After induction immunochemotherapy, peripheral blood stem cell (PBSC) collection was performed following mobilization with subcutaneous granulocyte colony-stimulating factor (G-CSF) at 10 μg/kg/day for five days. At the discretion of treating physicians, some patients also received a single intravenous dose of etoposide (375 mg/m^2^) for PBSC mobilization. For patients whose CD34^+^ cell count remained below 15 cells/mm^3^ after mobilization with G-CSF ± etoposide, plerixafor (0.24 mg/kg subcutaneously) was administered using a risk-adapted strategy as previously reported [18]. The collected PBSCs were infused during both the first ASCT and the subsequent tASCT. Conditioning regimens comprised high-dose melphalan (70 or 100 mg/m^2^/day for two days), administered alone or with busulfan (3.2 mg/kg/day for three days) [19]. The choice of conditioning regimen and maintenance therapy after tASCT was determined at the physician’s discretion. Of the 28 patients, 20 (71.4%) received maintenance therapy with either thalidomide or lenalidomide, with dosages adjusted based on individual factors such as estimated glomerular filtration rate and affordability. Supportive care, including bisphosphonates, antibiotics, and intravenous immunoglobulin, was provided according to institutional protocols, as detailed in previous reports [19, 20].

### Definition and Statistical analysis

Continuous variables are expressed as means ± standard deviation (SD) or standard error (SE), and categorical variables as frequencies or percentages. Response criteria were assessed according to International Myeloma Working Group guidelines [2]. Cytogenetic risk classification followed the Second Revision of the International Staging System (R2-ISS) [21]. The index date was defined as the date of PBSC infusion for tASCT. Neutrophil engraftment was defined as the first of three consecutive days with an absolute neutrophil count > 0.5 × 10^9^/L. Platelet engraftment was defined as the first of seven consecutive days with a platelet count > 30 × 10^9^/L without transfusion. Non-hematologic toxicities were assessed up to Day + 100 post-transplant and graded according to the National Cancer Institute Common Terminology Criteria for Adverse Events (NCI-CTCAE), version 5.0. Survival analyses, including progression-free survival (PFS) and overall survival (OS), were performed from the date of tASCT. Kaplan–Meier analysis was used to generate survival curves, and the log-rank test was used for comparisons. PFS was calculated from tASCT to disease progression or death, whichever occurred first. OS was defined as the time from tASCT to death from any cause or last follow-up. Median follow-up was calculated using the reverse Kaplan–Meier method. Normality of data distributions was assessed with the Shapiro–Wilk test. Paired t-tests were conducted to compare MRD frequencies before and after tASCT. Statistical analyses were performed using JAMOVI (version 2.6.13) and SPSS Statistics (version 29.0.2.0). Results were visualized with GraphPad Prism (version 10.4.0) and R Statistical Software (version 4.4.1).

## Results

### Patient characteristics

Seventeen patients (60.7%) were male, with a median age of 56.4 years (range, 43.3–67.0). Before tASCT, treatment responses were PR (*n* = 3, 10.7%), VGPR (*n* = 10, 35.7%), CR (*n* = 8, 28.6%), and sCR (*n* = 7, 25.0%). VTd was the most commonly used induction regimen, administered to 13 patients (46.4%). In the total cohort, 20 patients (71.4%) received post-transplant maintenance therapy: 16 received lenalidomide, and four received thalidomide. Baseline characteristics of the cohort are summarized in Table 1.

**Table 1:** Demographics of the entire study cohort

Characteristics	Total (*n* = 28)
Age at tASCT, median (range)	57.2 (40.6–67.0)
Gender, male, n (%)	17 (60.7)
Median time from ASCT to first MRD measurement, days, median (range)	100.5 (73.0–277.0)
Type of MM	
IgG, n (%)	18 (64.3)
IgA, n (%)	8 (28.6)
Light chain disease, n (%)	2 (7.1)
Type of light chain	
Kappa, n (%)	19 (67.9)
Lambda, n (%)	9 (32.1)
Cytogenetic status at diagnosis	
Standard risk	8 (28.6)
High risk, according to R2-ISS	16 (57.1)
del (17p)	10 (62.5)
t (4:14)	9 (56.3)
1q +	13 (81.3)
Unknown	4 (14.3)
Induction treatment regimen prior to initial ASCT	
VTd, n (%)	13 (46.4)
DVTd, n (%)	4 (14.3)
VRD, n (%)	11 (39.3)
Collection regimen for ASCT	
G-CSF alone, n (%)	8 (28.6)
Etoposide + G-CSF, n (%)	8 (28.6)
G-CSF + Plerixafor, n (%)	12 (42.9)
Pretreatment regimen for first ASCT	
High dose melphalan at a dose of 200 mg/m2, n (%)	20 (71.4)
High dose melphalan at a dose of 140 mg/m^2^, n (%)	4 (14.3)
High dose melphalan at a dose of 100 mg/m2, n (%)	1 (3.6)
High dose melphalan at a dose of 140 mg/m^2^, combination with busulfan 9.6 mg/kg, n (%)	3 (10.7)
Response status after initial ASCT (before tASCT)	
PR, n (%)	3 (10.7)
VGPR, n (%)	10 (35.7)
CR, n (%)	8 (28.6)
sCR, n (%)	7 (25.0)
Maintenance therapy after initial ASCT, yes, n (%)	5 (17.9)
ECOG at tASCT	
0, n (%)	9 (32.1)
1, n (%)	17 (60.7)
2, n (%)	2 (7.1)
Lactate dehydrogenase at tASCT	
High than upper normal limit	3 (10.7)
Within upper normal limit	25 (89.3)
eGFR at tASCT, mL/min/1.73m^2^ (median, range)	85.58 (8.72–127.2)
eGFR < 20 ml/min/1.73m^2^, n (%)	1 (3.6)
Pretreatment for tASCT	
High dose melphalan at a dose of 200 mg/m^2^, n (%)	18 (64.3)
High dose melphalan at a dose of 140 mg/m^2^, n (%)	4 (14.3)
High dose melphalan at a dose of 100 mg/m^2^, n (%)	2 (7.1)
High dose melphalan at a dose of 140 mg/m^2^, combined with busulfan 9.6 mg/kg, n (%)	4 (14.3)
Infused dose of CD34 + cells, median, × 10^6^/kg, (range))	4.97 (1.90–11.22)
Maintenance treatment after tASCT, yes, n (%)	20 (71.4)
Lenalidomide	16 (80.0)
Thalidomide	4 (20.0)

*Abbreviations: ASCT*, autologous stem cell transplantation; *tASCT,* tandem autologous stem cell transplantation; *VTd,* bortezomib-thalidomide-dexamethasone; *DVTd*, daratumumab-bortezomib-thalidomide-dexamethasone; *VRd,* bortezomib-lenalidomide-dexamethasone; *G-CSF,* Granulocyte-Colony Stimulating Factor; *eGFR,* estimated Glomerular Filtration Rate; *PR,* Partial Response; *VGPR,* Very Good Partial Response; *CR,* Complete Response; *sCR,* stringent complete response; *MRD,* minimal residual disease

### Treatment response, MRD and survival outcomes

**Fig. 2:**
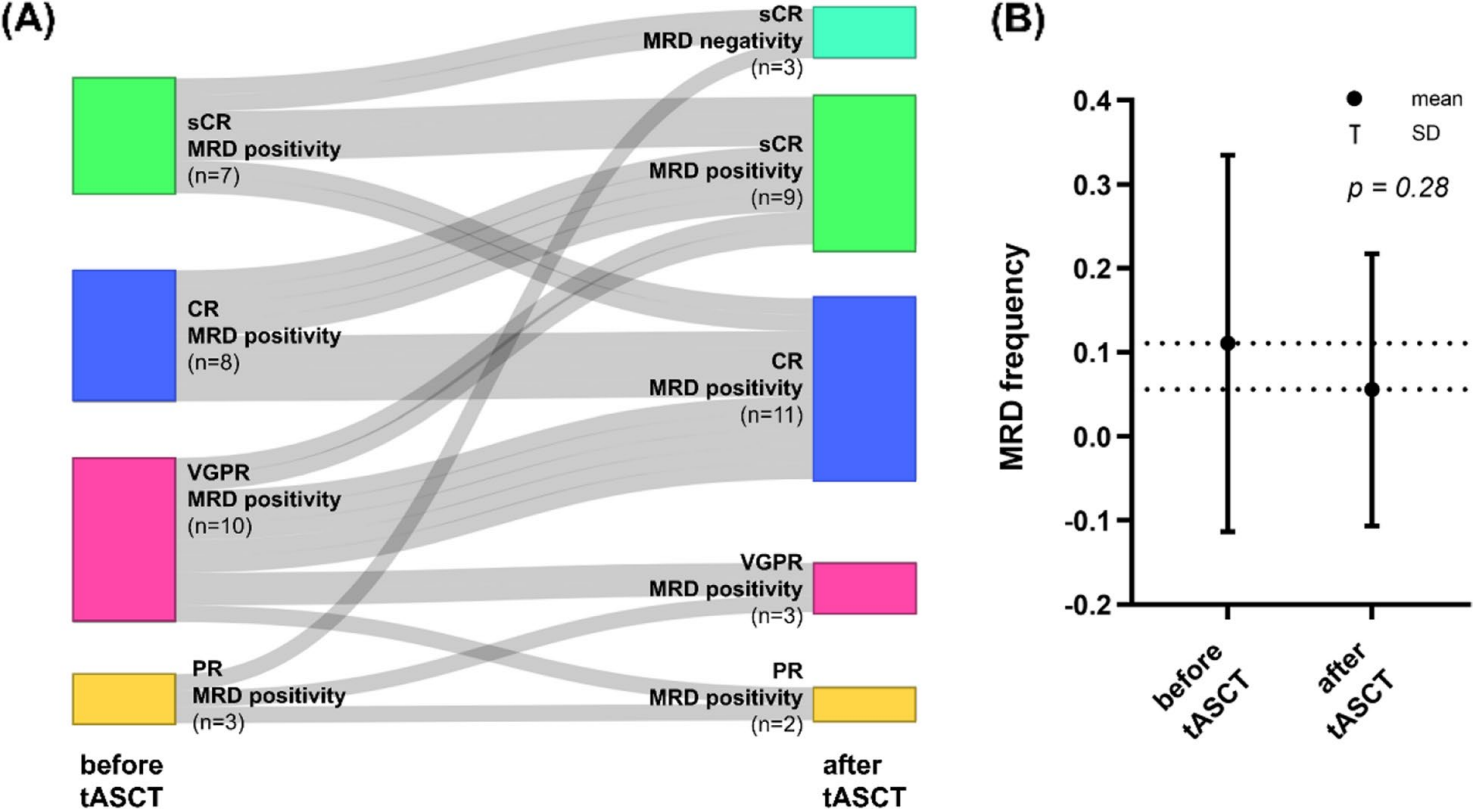
Response and MRD outcomes of the entire cohort. (**A**) Sankey diagram showing transitions of response status and MRD before and after tASCT. (**B**) Dynamics of MRD frequencies in the entire cohort, comparing pre- and post-tASCT measurements. *Abbreviations*: MRD, minimal residual disease; tASCT, tandem autologous stem cell transplantation

Following tASCT, 15 patients (53.6%) demonstrated an improved treatment response, 10 (35.7%) maintained their pre-tASCT response, and three (10.7%) experienced a decline. Beyond the IMWG response criteria, conversion from MRD positivity to MRD negativity was also considered an improvement. The best responses following tASCT were: sCR with MRD negativity (*n* = 3, 10.7%), sCR with MRD positivity (*n* = 9, 32.1%), CR with MRD positivity (*n* = 11, 39.3%), VGPR with MRD positivity (*n* = 3, 10.7%), and PR with MRD positivity (*n* = 2, 7.1%) (Fig. 2A).

**Fig. 3:**
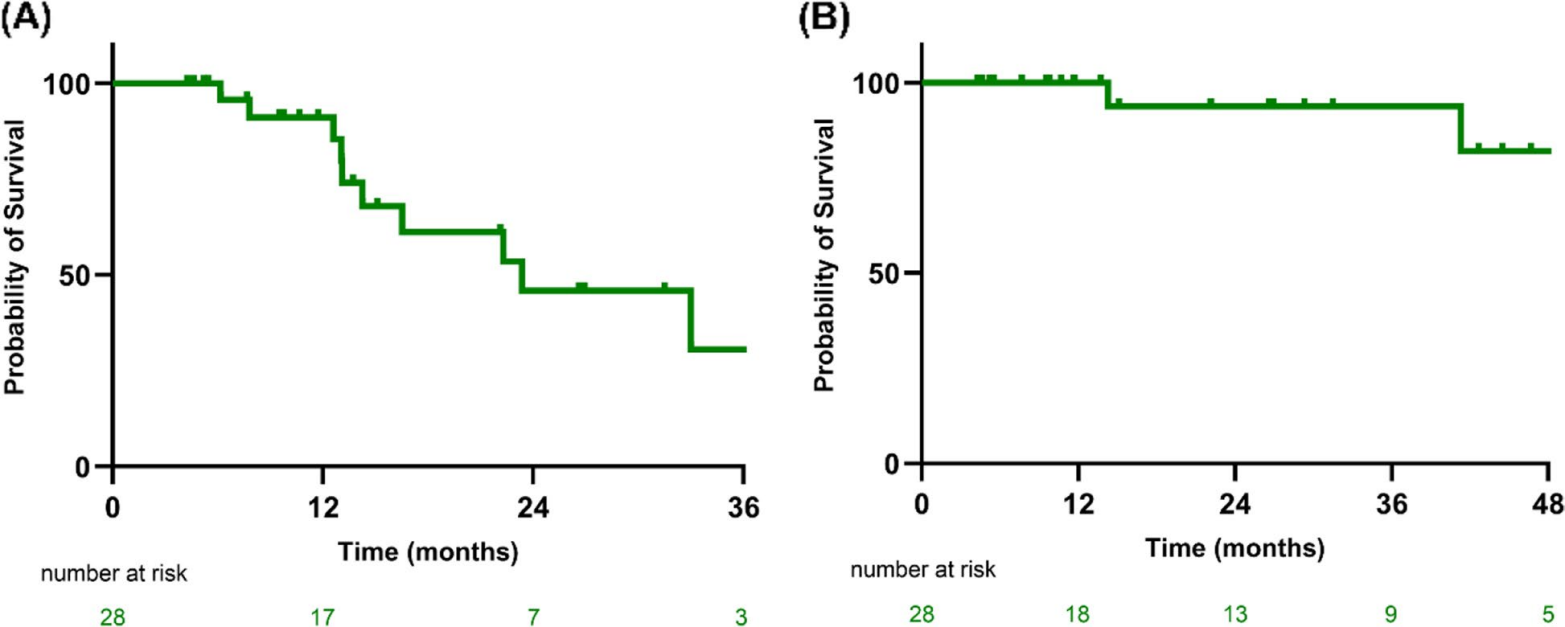
Survival outcomes of the entire cohort. (**A**) Progression-free survival (**B**) Overall survival

In the total cohort, mean MRD frequency decreased from 0.111% pre-tASCT (SD ± 0.224, SE ± 0.042) to 0.056% post-tASCT (SD ± 0.162, SE ± 0.031), with a mean reduction of 0.055% (*p* = 0.28; 95% CI, –0.047 to 0.157) (Fig. 2B). Twenty-three patients exhibited MRD reductions, including three who achieved MRD negativity. Conversely, five patients experienced increased MRD levels despite tASCT. With a median follow-up of 21.8 months (95% CI, 11.5–NA), the median PFS was 23.1 months (95% CI, 14.1–NA), while median OS was not reached (Fig. 3).

### Subgroup analysis according to MRD decrement

**Fig. 4:**
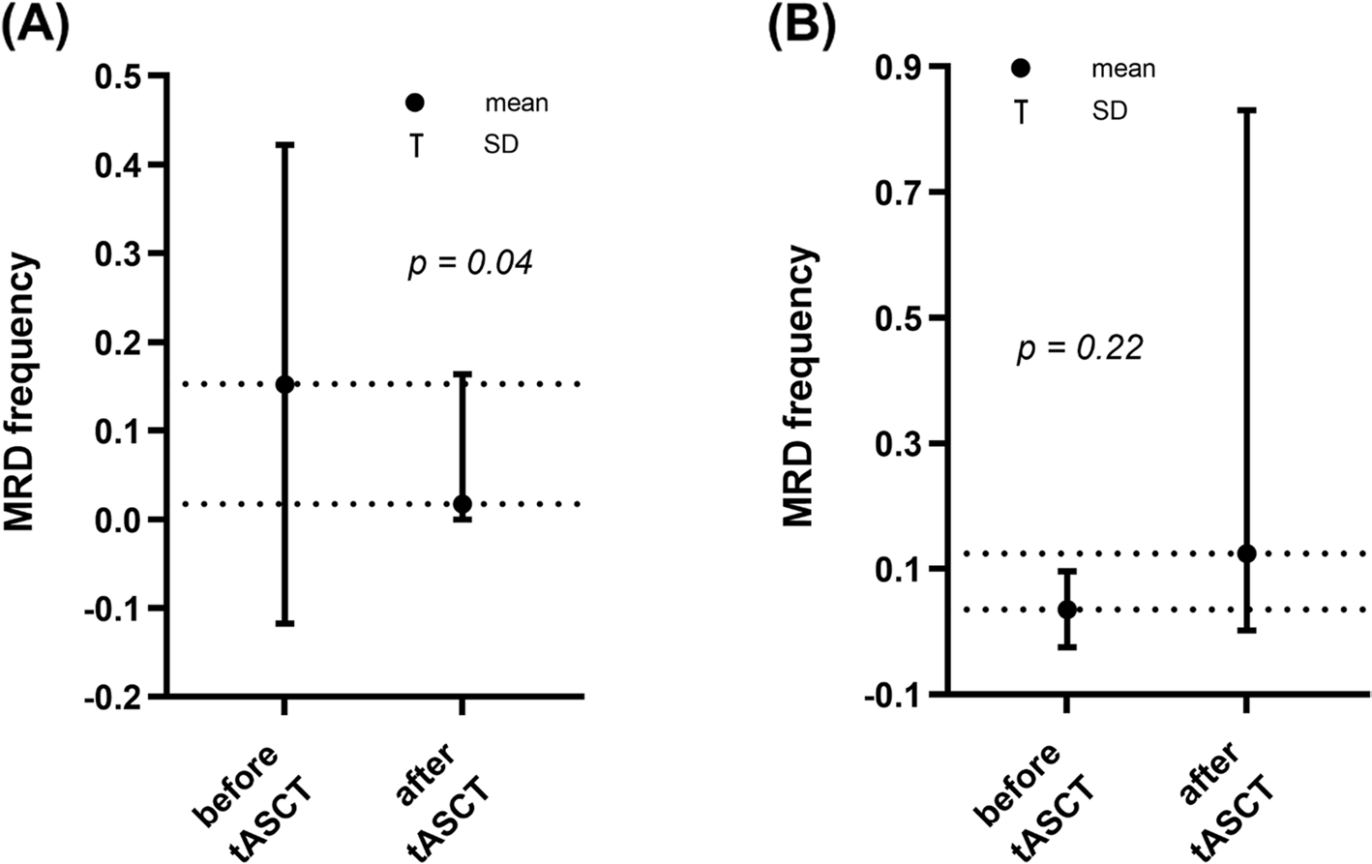
MRD dynamics in the extensive and modest clearance groups. (**A**) Changes in MRD frequencies before and after tASCT in the extensive clearance group, showing a significant reduction. (**B**) MRD frequency changes in the modest clearance group before and after tASCT. *Abbreviations*: MRD, minimal residual disease; tASCT, tandem autologous stem cell transplantation

Patients were stratified into two groups based on MRD reduction following tASCT: extensive MRD clearance (≥ 50% reduction, *n* = 18) and modest MRD clearance (< 50% reduction or increase, *n* = 10). In the extensive clearance group, three patients achieved MRD negativity, and the remaining 15 had MRD reductions ranging from half to nearly complete. Mean MRD frequency decreased significantly from 0.152% (SD ± 0.270, SE ± 0.064) to 0.017% (SD ± 0.039, SE ± 0.009), with a mean change of –0.135% (*p* = 0.04) (Fig. 4A). In the modest clearance group, five patients experienced increased MRD, while five had reductions of less than half. Mean MRD frequency increased from 0.036% (SD ± 0.061, SE ± 0.019) to 0.125% (SD ± 0.260, SE ± 0.082), with a mean change of + 0.089% (*p* = 0.22) (Fig. 4B).

**Fig. 5:**
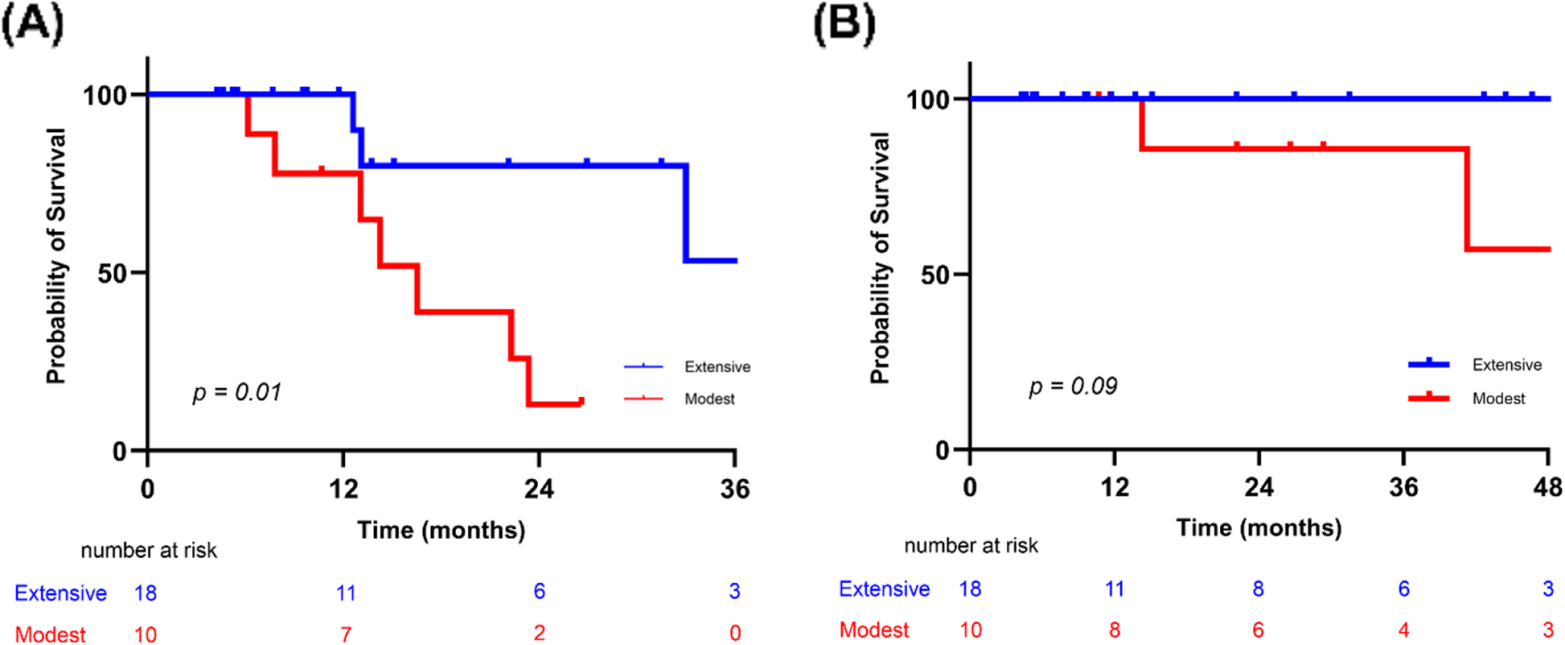
Survival outcomes in the extensive versus modest clearance groups. (**A**) Progression-free survival. (**B**) Overall survival. *Abbreviations*: Extensive, extensive MRD clearance group; Modest, modest MRD clearance group

For PFS, the extensive clearance group had significantly longer survival than the modest group (37.2 months [95% CI, 32.5–not reached] vs. 16.3 months [95% CI, 12.9–not reached], *p* = 0.01) (Fig. 5A). However, OS did not differ significantly between the two groups (*p* = 0.09) (Fig. 5B).

### Subgroup analysis according to post-transplant maintenance therapy

**Fig. 6:**
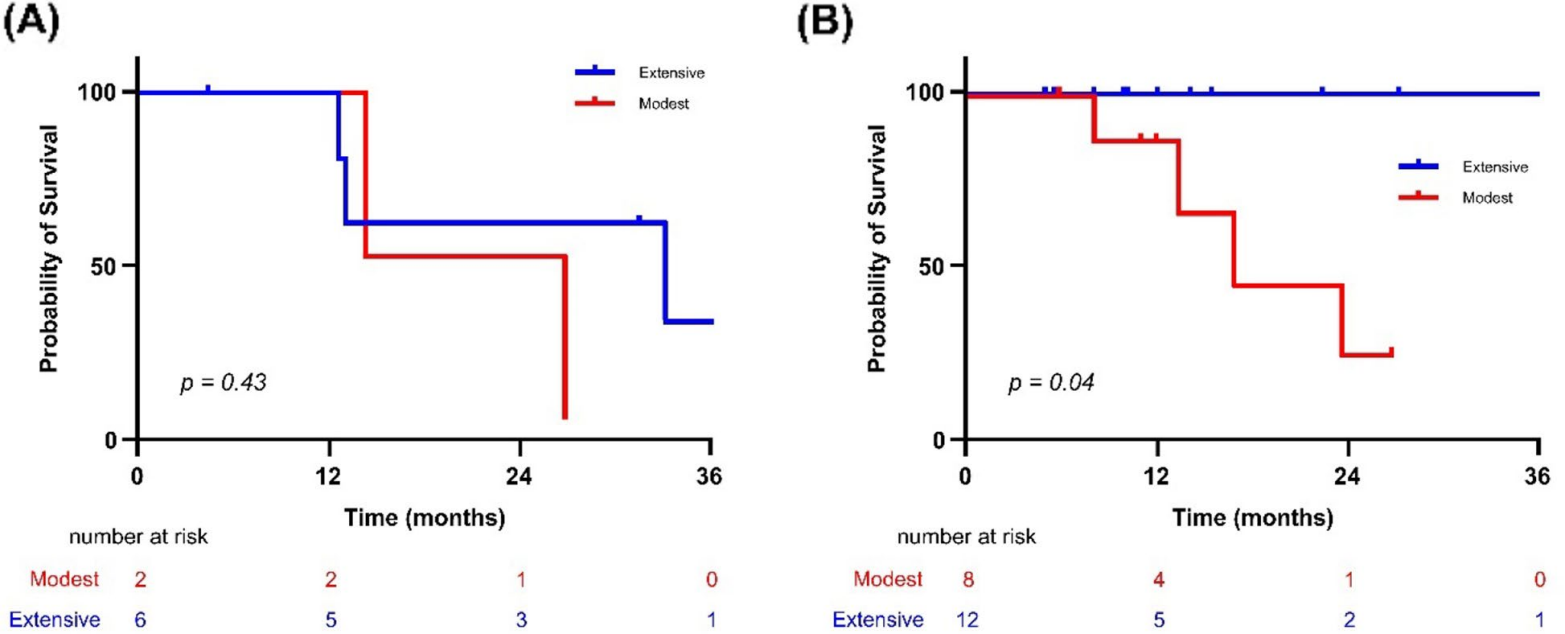
Progression-free survival (PFS) by MRD response status. (**A**) Patients without maintenance therapy. (**B**) Patients with maintenance therapy following tASCT. Abbreviations: Extensive, extensive MRD clearance group; Modest, modest MRD clearance group

We further explored whether MRD response influenced outcomes depending on maintenance therapy. Among patients without maintenance therapy (*n* = 8), PFS did not differ significantly between modest and extensive groups (20.5 vs. 33.0 months, *p* = 0.43). In contrast, among those who received maintenance therapy (*n* = 20), PFS was significantly longer in the extensive clearance group (45.8 vs. 16.5 months, *p* = 0.04) (Fig. 6).

### Engraftment and toxicity profile

**Fig. 7:**
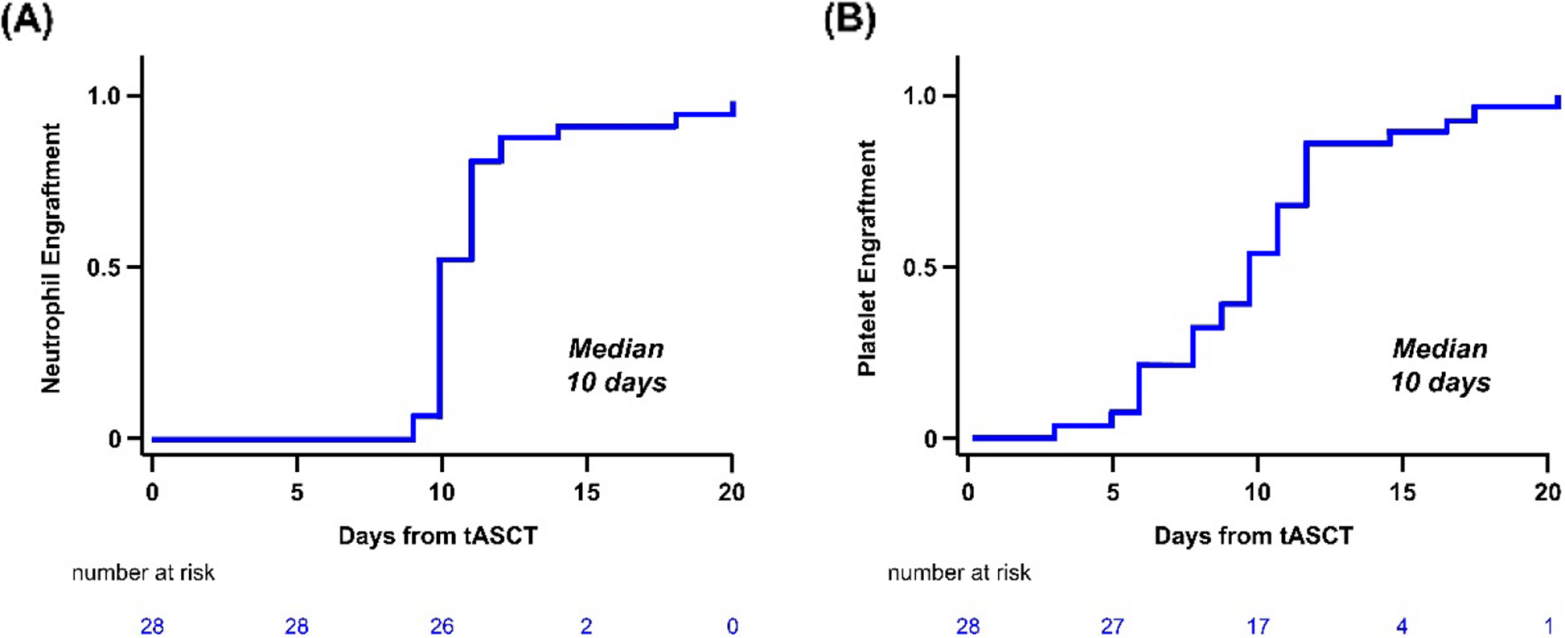
Cumulative incidence of (**A**) neutrophil and (**B**) platelet engraftment

No cases of graft failure or poor graft function were observed. Median time to neutrophil engraftment was 10 days (95% CI, 10–11), and median time to platelet engraftment was 10 days (95% CI, 9–12) (Fig. 7). Non-hematologic adverse events (AEs) are summarized in Table 2. The most common adverse event (AE) of any grade was nausea/vomiting (*n* = 24, 85.7%), followed by febrile neutropenia (*n* = 18, 64.3%) and psychiatric disturbance (*n* = 15, 53.6%). The most common grade ≥ 3 AE was febrile neutropenia (*n* = 18, 64.3%), followed by bacteremia (*n* = 7, 25.0%) and nausea/vomiting (*n* = 7, 25.0%). All AEs were effectively managed with supportive care (Table 2).

**Table 2:** Incidence of treatment-related toxicities

Adverse events	Total (*n* = 28)		
	All	G3	G4
Non-hematologic			
Febrile neutropenia	18 (64.3)	18 (64.3)	0 (0)
Infection, bacteremia	7 (25.0)	7 (25.0)	0 (0)
Nausea/Vomiting	24 (85.7)	7 (25.0)	0 (0)
Liver toxicity	9 (32.1)	1 (3.6)	0 (0)
Liver enzyme elevation	8 (28.6)	1 (3.6)	0 (0)
Bilirubinemia	3 (10.7)	0 (0)	0 (0)
Neuropathy	6 (21.4)	0 (0)	0 (0)
Renal toxicity	3 (10.7)	1 (3.6)	0 (0)
Veno-occlusive disease or sinusoidal obstruction syndrome	0 (0)	0 (0)	0 (0)
Psychiatric disturbance	15 (53.6)	0 (0)	0 (0)

*Abbreviations: G3,* Grade 3; *G4,* Grade 4

## Discussion

This exploratory study investigated the potential impact of tASCT in MM patients who remained MRD-positive after their initial ASCT. The extent of MRD reduction after tASCT varied among patients, reflecting the underlying heterogeneity in treatment response. When stratifying patients by the depth of MRD clearance, those with extensive MRD reduction experienced improved PFS. This effect appeared more pronounced among patients who also received maintenance therapy, suggesting that the prognostic relevance of MRD may be influenced by post-transplant treatment. While these findings indicate that tASCT may help deepen responses in selected MRD-positive patients, the results should be interpreted with caution because of the retrospective design and its inherent limitations.

MRD has emerged as a pivotal prognostic marker and surrogate endpoint in MM, providing a dynamic reflection of disease burden and response to therapy. Achieving MRD negativity has been consistently associated with significant improvements in PFS and OS, irrespective of baseline cytogenetic risk profiles [22, 23]. The prognostic utility of MRD has been validated across both newly diagnosed and relapsed/refractory MM, through large clinical trials [24, 25] as well as real-world evidence [7, 26]. However, patients who remain MRD-positive following first-line therapy constitute a high-risk subgroup with limited treatment options and poor outcomes. tASCT, by offering an additional high-dose chemotherapy intervention, presents a potential strategy to mitigate residual disease burden and improve responses [15]. In this context, our findings are consistent with results from the EMN02/HOVON95 trial, which demonstrated the value of intensified consolidation in achieving deeper responses and prolonging survival, especially among MRD-positive patients after frontline therapy [27]. By focusing on this high-risk group, our study suggests that tASCT can deepen MRD responses in selected patients and may represent one approach to addressing ongoing challenges in the treatment of MRD-positive MM. These observations support considering MRD status in clinical decision-making to guide individualized treatment strategies for patients with persistent disease after initial therapy.

While MRD negativity is an established treatment goal, our results raise the possibility that even partial reductions in MRD may have prognostic value. Patients with extensive MRD clearance—defined as > 50% reduction—showed a trend toward improved PFS compared with those with modest clearance. Although limited by sample size, this suggests that the depth of MRD reduction, even short of negativity, could still provide clinically relevant information. Stratifying patients based on MRD dynamics may guide treatment selection, helping clinicians determine who might benefit from additional consolidation or alternative therapy. Previous studies have predominantly focused on the achievement of MRD negativity as the primary binary endpoint, but emerging evidence supports the relevance of intermediate reductions. Our results align with studies reporting that incremental MRD reductions, even without full negativity, correlate with clinical outcomes [28]. Recognizing MRD as a continuous variable may provide a more nuanced approach to monitoring treatment response. Additionally, the observation that MRD reduction can attenuate the adverse effects of high-risk cytogenetics highlights the value of dynamic response evaluation beyond static baseline stratification [29].

For patients with persistent MRD positivity after first ASCT, tASCT may represent a viable option for achieving deeper responses. Our findings are consistent with the STAMINA trial, which reported improved PFS and OS in high-risk patients with cytogenetic abnormalities who underwent tASCT [30]. Building on this evidence, our study demonstrates that tASCT can reduce MRD levels, with clinical benefit observed in those achieving extensive clearance. These findings suggest that tASCT could play a role in treatment strategies for MRD-positive MM, particularly in contexts where alternative intensification approaches, such as consolidation with novel agents, are less available or effective. Incorporating MRD as a stratification tool may enhance the precision of treatment planning and support the broader shift toward personalized medicine in MM. By monitoring MRD dynamics to guide therapy, clinicians may better identify patients most likely to benefit from tASCT, thereby improving outcomes in this high-risk group. These findings may contribute to the growing evidence base supporting the use of MRD as both a prognostic marker and a tool for refining treatment approaches in MM.

Although this study did not directly evaluate the safety of tASCT, toxicity was generally manageable, with no graft failure or delayed engraftment. Non-hematologic adverse events were consistent with prior reports of high-dose therapy and ASCT. However, given the intensity of tASCT, further research is warranted to evaluate its full risk–benefit profile in MRD-positive patients.

Several limitations should be acknowledged. First, the small sample size and retrospective design limit the generalizability of our findings and underscores the need for larger, prospective studies. Second, heterogeneity in conditioning regimens and induction/maintenance strategies may have introduced confounders. Third, although MRD reduction was used as a surrogate endpoint, the optimal threshold for meaningful MRD clearance remains undefined. Finally, variability in MRD assessment timing and methodology, inherent to retrospective analyses, may also have influenced the results.

This study highlights the potential of tASCT to deepen responses in MM patients with persistent MRD positivity after initial ASCT. By demonstrating the prognostic relevance of MRD reduction, our findings emphasize the importance of integrating MRD dynamics into treatment planning. However, these findings should be considered hypothesis-generating. Prospective studies with larger cohorts are needed to validate the role of MRD-guided tASCT and refine strategies for this high-risk population.

## Data Availability

No datasets were generated or analysed during the current study.
